# Untargeted urine metabolomics reveals dynamic metabolic differences and key biomarkers across different stages of Alzheimer’s disease

**DOI:** 10.3389/fnagi.2025.1530046

**Published:** 2025-01-27

**Authors:** Xiaoya Feng, Shenglan Zhao

**Affiliations:** ^1^Department of Neurology, Shandong Provincial Third Hospital, Jinan, China; ^2^Department of Psychiatry and Psychology, Shandong Provincial Third Hospital, Jinan, China

**Keywords:** Alzheimer’s disease, mild cognitive impairment, urine metabolomics, biomarkers, key biomarkers

## Abstract

**Background:**

Alzheimer’s disease (AD) is a progressive neurodegenerative disorder, with mild cognitive impairment (MCI) often serving as its precursor stage. Early intervention at the MCI stage can significantly delay AD onset.

**Methods:**

This study employed untargeted urine metabolomics, with data obtained from the MetaboLights database (MTBLS8662), combined with orthogonal partial least squares-discriminant analysis (OPLS-DA) to examine metabolic differences across different stages of AD progression. A decision tree approach was used to identify key metabolites within significantly enriched pathways. These key metabolites were then utilized to construct and validate an AD progression prediction model.

**Results:**

The OPLS-DA model effectively distinguished the metabolic characteristics at different stages. Pathway enrichment analysis revealed that Drug metabolism was significantly enriched across all stages, while Retinol metabolism was particularly prominent during the transition stages. Key metabolites such as Theophylline, Vanillylmandelic Acid (VMA), and Adenosine showed significant differencesdifferencesin the early stages of the disease, whereas 1,7-Dimethyluric Acid, Cystathionine, and Indole exhibited strong predictive value during the MCI to AD transition. These metabolites play a crucial role in monitoring AD progression. Predictive models based on these metabolites demonstrated excellent classification and prediction capabilities.

**Conclusion:**

This study systematically analyzed the dynamic metabolic differences during the progression of AD and identified key metabolites and pathways as potential biomarkers for early prediction and intervention. Utilizing urinary metabolomics, the findings provide a theoretical basis for monitoring AD progression and contribute to improving prevention and intervention strategies, thereby potentially delaying disease progression.

## Highlights:

• A comprehensive evaluation of dynamic metabolic differences across different stages of Alzheimer’s disease (AD) progression was conducted using untargeted urine metabolomics combined with orthogonal partial least squares discriminant analysis (OPLS-DA) models.
• Key metabolic pathways, such as Drug metabolism and Retinol metabolism, were identified as playing crucial roles in predicting AD progression.
• Key metabolites for dynamic monitoring and prediction of AD progression were identified. Theophylline, Vanillylmandelic Acid (VMA), and Adenosine showed potential as predictive biomarkers in the early stages of AD (from cognitively normal to mild cognitive impairment), while 1,7-Dimethyluric Acid, Cystathionine, and Indole demonstrated stronger predictive power at the critical transition point of AD (MCI).
• An AD progression prediction model was constructed, showing strong classification ability of key metabolites for disease staging.

## Introduction

Alzheimer’s disease (AD) is a progressive neurodegenerative disorder primarily affecting individuals over the age of 60, with a global prevalence estimated at 57 million. Symptoms of AD include memory loss, cognitive decline, and behavioral differences (Nichols and Vos, 2022; Zvìøová M, 2019). Despite extensive research, the precise etiology of AD is still not well understood, and its complex pathogenesis has hindered the development of effective treatments (Loera-Valencia et al., 2019). In recent years, metabolomics has provided a new perspective for identifying diagnostic biomarkers for AD. Metabolomics is a high-throughput technique for analyzing small molecules within biological systems, offering insights into potential disease mechanisms by examining differences in metabolites in bodily fluids (Clish, 2015). Increasing evidence suggests that metabolic dysregulation plays a key role in the progression of AD (Rojas-Gutierrez et al., 2017). Existing metabolomics studies have identified a range of potential AD biomarkers through the analysis of blood and cerebrospinal fluid (CSF) (Barthélemy et al., 2024; Schweickart et al., 2024). However, blood samples are susceptible to external factors, may compromise diagnostic accuracy, and the invasive nature of CSF collection limits its broader clinical application. In contrast, urine metabolomics, due to its non-invasive nature and ease of sample collection, presents significant potential for the early diagnosis of AD (Zhang et al., 2022).

Mild cognitive impairment (MCI), often recognized as “MCI due to AD”, is a precursor stage to AD (Jack et al., 2018; Liss et al., 2021). It is estimated that 21% of individuals with MCI will develop mild AD by the age of 65 (Davis et al., 2018). Although not all MCI cases progress to AD, with some remaining stable or even reverting to normal cognition, it is generally observed that nearly all MCI cases directly attributable to underlying Alzheimer’s pathology will eventually progress to full-blown AD (Cardoso et al., 2020; Jack et al., 2018; Pandya et al., 2016). Typical symptoms of MCI include mild memory impairment and cognitive decline, but these are not severe enough to significantly affect daily life (Anderson, 2019; Petersen et al., 2018). In its late stages, AD is ultimately fatal (Seo and Holtzman, 2024). Therefore, identifying biomarkers for early MCI and its progression to AD could greatly improve early detection rates and slow disease progression. Although previous studies have identified potential metabolites related to AD and MCI through urine metabolomics (Wang et al., 2023; Yilmaz et al., 2020), there is currently a lack of comprehensive comparative analysis of the transition from cognitively normal (CN) to MCI and AD, particularly concerning metabolite differences and associated metabolic pathways at specific stages.

Thus, this study aims to systematically analyze the metabolic dynamics across different stages of AD progression, with a focus on metabolic differences emerging at MCI as a critical juncture in the development of AD. Utilizing untargeted metabolomics combined with orthogonal partial least squares-discriminant analysis (OPLS-DA), the study seeks to identify key metabolites and associated metabolic pathways closely linked to the dynamic differences in AD progression. This research provides new insights into the pathological mechanisms of AD and offers important targets and theoretical support for early prediction and intervention strategies for AD.

## Materials and methods

### Data source and metabolite identification

The metabolomics data used in this study were obtained from the MetaboLights database (MTBLS8662). MetaboLights is a global database for metabolomics studies including the raw experimental data and the associated metadata (Yurekten et al., 2024). Our dataset consisted of urine metabolomics data from 162 participants aged 50 and above. Based on cognitive tests and medical history evaluations, participants were divided into three groups: the AD group (57 participants), the MCI group (43 participants), and the CN group (62 participants). AD was clinically diagnosed according to the 2011 National Institute on Aging-Alzheimer’s Association (NIA-AA) criteria (McKhann et al., 2011). MCI was defined using the same 2011 NIA-AA diagnostic criteria (Albert et al., 2011). CN controls were defined as individuals who performed within normal limits on standardized neuropsychological tests and had no significant cognitive concerns or complaints during the structured interview. Participants in the study were assessed for various comorbidities, including hypertension, diabetes, hyperlipidemia, heart diseases, and cerebrovascular diseases, along with demographic data such as age, gender, and education level, as outlined in Table 1. To control potential effects of hydration on metabolite concentrations, 200 μL of urine samples were placed in centrifuge tubes and resuspended with prechilled 80% methanol, followed by vortexing. After incubation on ice for 5 min, the samples were centrifuged at 15,000 g for 20 min at 4°C. A portion of the supernatant was diluted with LC–MS grade water to achieve a final concentration of 53% methanol. The samples were then transferred to new centrifuge tubes and centrifuged again for 20 min at 15,000 g at 4°C. The supernatant was collected and analyzed using an LC–MS/MS system (Wang et al., 2023).

**TABLE 1:** Basic information and neuropsychological assessment.

	AD (*n* = 57)	MCI (*n* = 43)	CN (*n* = 62)	*P*
Age (median, P25, P75)	79 (72.5, 82)	74 (68, 78)	70(63.75, 73.5)	<0.001^a^
Gender (male/female)	27/30	14/29	22/40	0.253
Smoking (yes/no)	15/42	8/35	10/52	0.366
Hypertension (yes/no)	22/35	15/28	31/31	0.247
Diabetes (yes/no)	11/46	5/38	16/46	0.199
Hyperlipidemia (yes/no)	23/34	23/20	35/27	0.186
Heart diseases (yes/no)	16/41	10/33	10/52	0.288
Cerebrovascular diseases (yes/no)	14/43	8/35	11/51	0.618
Family history (yes/no)	10/47	7/36	13/49	0.808
APOE (ε4 carrier/non-carrier)	27/30	21/22	14/48	0.005^a^^b^
**Global cognition**				
MMSE	15 (9.5, 19)	24 (22, 26)	27 (26, 28)	<0.001^a^^b^
MoCA	7 (4, 12)	18 (13.75, 21)	23 (21, 26)	<0.001^a^^b^
**Visuospatial ability**				
CDT	5 (1, 17)	22 (15, 28)	26 (24, 30)	<0.001^a^^b^
RCFT	0 (0, 0)	2 (0, 8.5)	11 (5, 17)	<0.001^a^^b^
**Language**				
BNT	10 (7, 16.25)	19 (15, 23)	26 (23, 27.25)	<0.001^a^^b^
VFT	15 (8.75, 21)	26 (23, 34)	40 (34, 49)	<0.001^a^^b^

^a^Indicates significant differences between AD and CN (Bonferroni-corrected *P* < 0.05).

^b^Indicates significant differences between MCI and CN (Bonferroni-corrected *P* < 0.05).

Peak alignment, selection, and quantification were conducted for both positive and negative ion modes were performed using MZmine3 software, ensuring the independence of each ion mode dataset. The raw data was reprocessed using untargeted metabolomics analysis, followed by quality control (QC). Metabolites with a coefficient of variation (CV) over 30% in QC samples were excluded to ensure data reliability. Metabolite identification was conducted using the GNPS platform, with results cross-referenced against multiple public databases (including HMDB, CASMI, MSMLS, MONA, NIH, and SCIE) to ensure accuracy. To handle missing data, we used the K-nearest neighbors (KNN) algorithm for imputation. Specifically, missing values were imputed by considering 10% of the sample size from each sample group. The KNN algorithm uses the closest available samples to estimate and fill in the missing data points.

### Differential metabolite screening and classification

To identify differential metabolites, partial least squares discriminant analysis (PLS-DA) was performed on the entire sample set using the Scikit-learn library in Python 3.12 to assess the distribution characteristics of the data. PLS-DA scatter plots were generated to visualize the distribution of samples. The samples were divided into three comparison groups representing different stages of AD progression: the CN-AD group, CN-MCI group, and MCI-AD group.

The OPLS-DA model was constructed using the Numpy library, and a 200-time permutation test was conducted to evaluate the robustness and reliability of the model. Differential metabolites were identified using the variable importance in projection (VIP > 1) from the first component of the OPLS-DA model, Wilcoxon rank-sum test *p*-values (*p* < 0.05), and fold change values (FC < 0.8 or FC > 1.25). Volcano plots were used to visually display the overall distribution of differential metabolites, showing the relationship between their significance and FC values.

### Pathway enrichment and construction of a key metabolite prediction model

The differential metabolites identified were annotated using the Kyoto Encyclopedia of Genes and Genomes (KEGG) and the Human Metabolome Database (HMDB). Based on these annotations, pathway enrichment analysis was performed. A decision tree approach was then applied to select the differential metabolites involved in these key metabolic pathways. A logistic regression model was constructed based on these key metabolites to predict AD progression. Upon constructing the model, ROC curves were generated, and AUC values were calculated to evaluate the model’s classification ability. A 200-time random sampling test was conducted to ensure model robustness, while R^2^ and Q^2^ values were used to assess the reliability and predictive power of the model.

### Statistical analysis

All statistical analyses were performed using relevant libraries in Python 3.12, including Scipy and Scikit-learn. Normality tests were first conducted to assess the distribution of the data. For normally distributed data, Hotelling’s T^2^ test was used to evaluate intergroup differences; for non-normally distributed data, the Mann-Whitney U test was applied.

## Results

### Metabolite identification results

**FIGURE 1:**
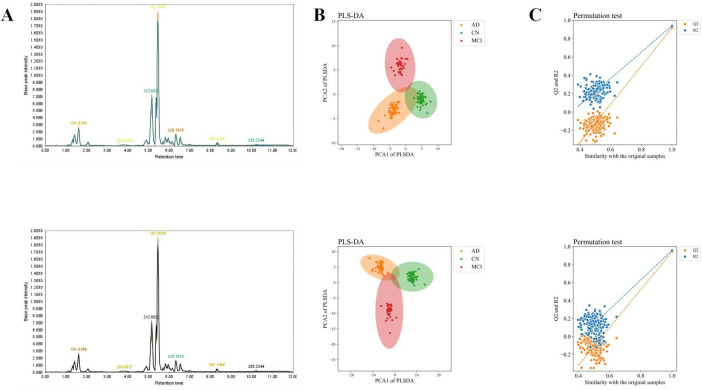
Total ion chromatogram, overall sample PCA scatter plot, and overall sample loading plot. **(A)** Total ion chromatogram of QC samples. **(B)** PLS-DA scatter plot of overall samples. **(C)** Loading plot of overall samples. The upper row represents negative ion mode, and the lower row represents positive ion mode.

After QC, a total of 1,128 metabolites were detected, with 325 metabolites identified in negative ion mode and 803 metabolites in positive ion mode. The total ion chromatograms for all QC samples demonstrated high consistency in response intensity and retention time, indicating reliable data sources (Figure 1A). The PLS-DA results demonstrated robust modeling metrics for both negative and positive ion modes (Figure 1B). Additionally, permutation testing confirmed the robustness of the model, verifying that the group distinctions are statistically significant (Figure 1C).

### Differential metabolite screening results

**FIGURE 2:**
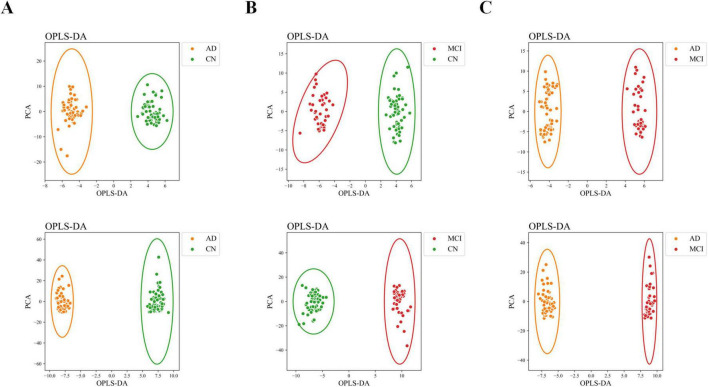
OPLS-DA scatter plot. **(A)** OPLS-DA scatter plot of the CN-AD group. **(B)** OPLS-DA scatter plot of the CN-MCI group. **(C)** OPLS-DA score plot of the MCI-AD group. The upper row represents negative ion mode, and the lower row represents positive ion mode.

**FIGURE 3:**
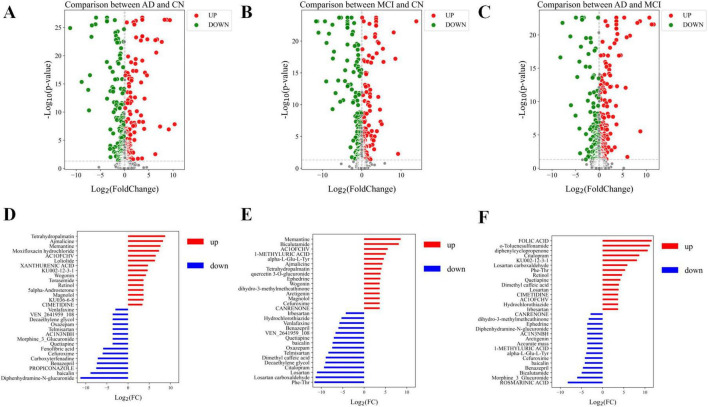
Volcano plots of differential metabolites and bar charts of differential metabolites. **(A)** Volcano plot of differential metabolites in the CN-AD group. **(B)** Volcano plot of differential metabolites in the CN-MCI group. **(C)** Volcano plot of differential metabolites in the MCI-AD group. **(D)** Bar chart of the top 15 upregulated and downregulated differential metabolites in the CN-AD group. **(E)** Bar chart of the top 15 upregulated and downregulated differential metabolites in the CN-MCI group. **(F)** Bar chart of the top 15 upregulated and downregulated differential metabolites in the MCI-AD group. In the volcano plot, red dots represent metabolites that are significantly upregulated (*p* < 0.05), green dots indicate metabolites that are significantly downregulated (*p* < 0.05), and gray dots denote metabolites with no significant differential expression.

The OPLS-DA model effectively distinguished the three comparison groups (CN-AD, CN-MCI, and MCI-AD), with clear separation observed between the groups in both positive and negative ion modes (Figures 2A–C). Permutation tests further validated the robustness and reliability of the model (Supplementary Figures S1A–C). The predictive performance of the original model was significantly surpassed that of the permutation model, with R^2^ values exceeding 0.9 and Q^2^ values exceeding 0.7 in both the positive and negative ion modes (Tables 2, 3). VIP plots demonstrated the importance of differential metabolites in model classification (Supplementary Figures S2A–C). The volcano plots presented the differential metabolites identified in both positive and negative ion modes (Figures 3A–C), while the top 15 upregulated and downregulated metabolites in each group (Figures 3D–F).

**TABLE 2:** R2 and Q2 values and number of differential metabolites in the OPLS-DA model for each group in the negative ion model.

Group	R2	Q2	Number of differential metabolites screened
CN-AD	0.98	0.77	53
CN-MCI	0.98	0.79	56
MCI-AD	0.99	0.71	45

**TABLE 3:** R2 and Q2 values and number of differential metabolites in the OPLS-DA model for each group in the positive ion model.

Group	R2	Q2	Number of differential metabolites screened
CN-AD	0.99	0.74	119
CN-MCI	0.98	0.79	125
MCI-AD	0.99	0.72	115

### Metabolic pathways associated with AD progression

**FIGURE 4:**
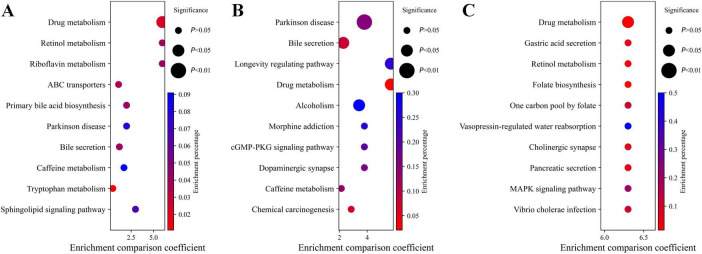
Pathway enrichment analysis. **(A)** Pathway enrichment analysis for the CN-AD group. **(B)** Pathway enrichment analysis for the CN-MCI group. **(C)** Pathway enrichment analysis for the MCI-AD group. **(D)** Mechanism diagram illustrating the relationships at different stages of AD.

Enrichment analysis revealed significant enrichment of pathways such as Drug metabolism, Retinol metabolism, and Riboflavin metabolism in the CN-AD group (Figure 4A); Drug metabolism, Longevity Regulation Pathways, and Parkinson’s Disease pathways were enriched in the CN-MCI group (Figure 4B); and Drug metabolism, Gastric Acid Secretion, Cholinergic Synapse, and Retinol metabolism pathways were enriched in the MCI-AD group (Figure 4C). Notably, Drug metabolism was enriched in all three groups, while Retinol metabolism was enriched in both the AD-CN and AD-MCI groups.

### Identification of key metabolites in AD progression

**FIGURE 5:**
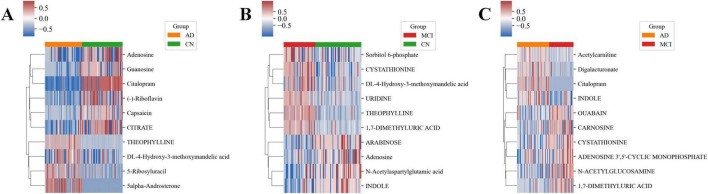
Heatmaps of key metabolites in each group. **(A)** Heatmap of key metabolites in the CN-AD group. **(B)** Heatmap of key metabolites in the CN-MCI group. **(C)** Heatmap of key metabolites in the MCI-AD group.

The decision tree method identified 10 key metabolites for each group (Supplementary Figures S3A–C). In the CN-AD group, 5α-Androsterone and Theophylline were significantly upregulated, while Adenosine and Capsaicin were significantly downregulated (Figure 5A). In the CN-MCI group, Theophylline and 1,7-Dimethyluric Acid were significantly upregulated, while Adenosine and Arabinose were significantly downregulated (Figure 5B). In the MCI-AD group, Citalopram and Digalacturonate were significantly upregulated, while 1,7-Dimethyluric Acid and N-Acetylglucosamine were significantly downregulated (Figure 5C). Box plots showed the significant differences in key metabolites between the comparison groups (Supplementary Figures S4A–C).

Additionally, several metabolites exhibited consistent or opposing differences across different groups. For example, Vanillylmandelic Acid (VMA, DL-4-Hydroxy-3-Methoxymandelic Acid) was significantly upregulated in both the CN-AD and CN-MCI groups, while Adenosine was significantly downregulated in both groups. 1,7-Dimethyluric Acid and Cystathionine were significantly upregulated in the CN-MCI group but significantly downregulated in the MCI-AD group. Conversely, Indole was significantly downregulated in the CN-MCI group but significantly upregulated in the MCI-AD group.

### Construction and validation of AD progression prediction model

**FIGURE 6:**
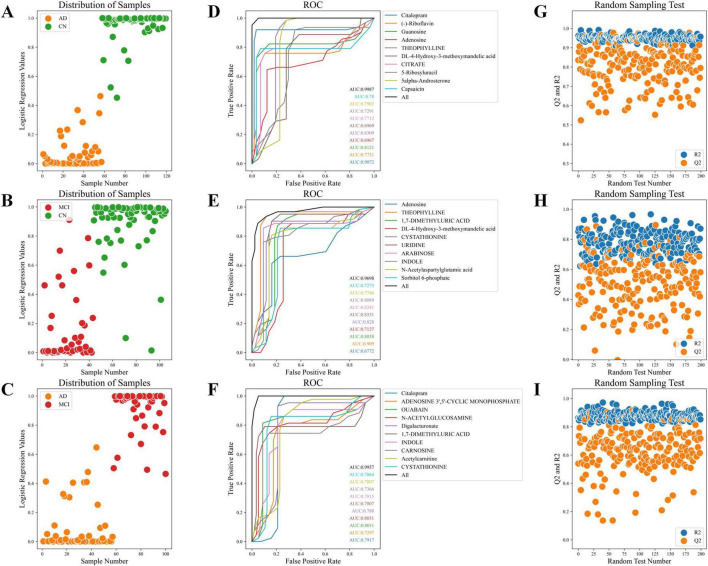
Sample distribution, ROC curves, and random sampling tests. **(A)** Sample distribution plot of the CN-AD group. **(B)** Sample distribution plot of the CN-MCI group. **(C)** Sample distribution plot of the MCI-AD group. **(D)** ROC curve of the CN-AD group. **(E)** ROC curve of the CN-MCI group. **(F)** ROC curve of the MCI-AD group. **(G)** Random sampling test for the CN-AD group. **(H)** Random sampling test for the CN-MCI group. **(I)** Random sampling test for the MCI-AD group.

The sample distribution plots demonstrated the excellent classification ability of the AD progression prediction model based on key metabolites across different groups (Figures 6A–C). The ROC curve showed outstanding predictive power of the model in distinguishing different stages of AD, with combined AUC values of 0.999, 0.940, and 0.996 for the CN-AD, CN-MCI, and MCI-AD groups, respectively (Figures 6D–F). The results from the random sampling tests showed that the model had good predictive accuracy (Figures 6G–I).

## Discussion

This study employed untargeted urine metabolomics combined with OPLS-DA to analyze metabolic differences across various stages of AD, identifying significantly enriched metabolic pathways and key metabolites at each stage. Based on these findings, an AD progression prediction model was constructed. Previous studies have primarily focused on blood and brain metabolism in MCI and AD patients (Pillai et al., 2023; Salmon et al., 2024), while urine metabolomics research remains relatively scarce and preliminary (Wang et al., 2023; Yilmaz et al., 2020). The innovation of this study lies in its systematic analysis of urine metabolic differences across all stages of AD, revealing differences in key metabolites and pathways with significant potential for predicting AD progression.

Our study included 162 participants categorized into AD, MCI, and CN groups. We observed significant age differences: median ages were 79 for AD, 74 for MCI, and 70 for CN groups (*P* < 0.001). Gender distribution was consistent across groups (*P* = 0.253), ensuring gender neutrality in our findings. Lifestyle and health factors such as smoking, hypertension, diabetes, hyperlipidemia, heart conditions, cerebrovascular diseases, and family history of AD showed no significant differences across groups, minimizing their impact as confounders. Notably, the APOE ε4 allele was significantly more prevalent among AD patients (*P* = 0.005). Cognitive function, assessed through MMSE and MoCA scores, deteriorated progressively from CN to AD (*P* < 0.001), confirming the expected clinical trajectory.

The study corroborates and extends the understanding of drug metabolism and retinol metabolism pathways, which are crucial in predicting AD progression. While drug metabolism pathways are known to be significantly enriched in AD, impacting drug clearance, liver metabolism, and neurotransmitter balance (Dhurjad et al., 2022; Monteiro et al., 2023; Zhao et al., 2021), our findings provide further insight into the dynamic alterations of these pathways across various stages of the disease. Initially, in the early stages from cognitively normal (CN) to mild cognitive impairment (MCI), the impact of drug metabolism pathways appears relatively minor. However, even at this phase, we observe stability in the levels of drug metabolites such as Citalopram, indicating that drug metabolism remains functionally robust in MCI patients. This phase also marks the onset of subtle changes in the activity of metabolic enzymes such as CYP2C19, CYP2D6, and CYP3A4, which are involved in the N-demethylation of Citalopram to demethylcitalopram (Rao, 2007). As patients transition from MCI to AD, there is a pronounced shift in drug metabolism pathways, characterized by increased variability and altered levels of drugs like Codeine. This alteration reflects significant metabolic dysfunction, with Codeine’s metabolism through CYP2D6 to morphine—a potent opioid—undergoing substantial changes (Ashraf et al., 2024). These changes mirror the physiological shifts associated with advancing AD and suggest a significant impact on drug clearance rates, liver metabolism, and overall pharmacokinetics. These findings emphasize how the progression of AD increasingly compromises drug metabolism, which could significantly impact the pharmacodynamics and therapeutic outcomes of medications used in this population. Such insights suggest that monitoring drug metabolism pathways could serve as an early warning system for AD progression, assisting in identifying high-risk individuals and enhancing opportunities for timely intervention and treatment. Retinol metabolism played a key role in predicting AD progression.

Retinol and its derivatives are vital for several brain health-related processes, including neuronal differentiation, synaptic plasticity, and gene expression regulation (Dumetz et al., 2022; Moramarco and McCaffery, 2023). Notably, vitamin A, a primary form of retinol, significantly influences AD pathogenesis by modulating the deposition and clearance of amyloid-beta plaques, central to AD pathology (Chen et al., 2021). This connection underscores retinol’s potential impact on the underlying neurodegenerative processes of AD. Previous studies have linked Retinol metabolism to neuroprotection and antioxidant processes, suggesting its association with cognitive decline in AD patients (Wołoszynowska-Fraser et al., 2020). The present study further indicates that Retinol metabolism is significantly enriched during the transition from MCI to AD, possibly playing a critical role in this process. However, it is important to note that previous study (Bourdel-Marchasson, 2001) reported low plasma retinol concentrations in AD patients, which seems to contradict our findings. This discrepancy may be attributed to differences in the biological matrices (plasma vs. urine) used for measurements. The difference in retinol levels between plasma and urine can be due to the matrices used for measurement, where urine levels reflect the body’s excretion and metabolic processing rather than immediate bioavailability. The enrichment of retinol in urine could suggest the presence of compensatory mechanisms or adaptive responses aimed at maintaining retinol homeostasis, potentially due to decreased bioavailability in plasma. This increase in metabolic activity might be the body’s attempt to regulate or respond to the neurodegenerative processes inherent in AD progression. Therefore, the observed alterations in Retinol metabolism pathways may not only serve as a monitor for the transition from MCI to AD but also highlight potential targets for future interventions aimed at modulating this pathway to benefit AD patients.

This study also identified key metabolites for dynamically monitoring and predicting AD progression. Among these, Theophylline, VMA, and Adenosine demonstrated potential as early predictive markers during the initial stages of AD, while 1,7-Dimethyluric Acid, Cystathionine, and Indole exhibited stronger predictive power at the key transition point of AD progression (MCI). In the early stages of AD, the differential expression of Theophylline, VMA, and Adenosine were more pronounced, indicating their potential as early biomarkers. Theophylline, a xanthine derivative, has been shown to have anti-inflammatory and immunomodulatory properties, protecting neurons from inflammatory damage through the regulation of the Adenosine receptor (Janitschke et al., 2021). It is noteworthy that our results indicate the presence of caffeine metabolism, suggesting that Theophylline could potentially be a byproduct of this process (Monteiro et al., 2016). However, while Theophylline is a known metabolite of caffeine, it is important to clarify that our study does not determine whether the caffeine originates from dietary consumption or pharmaceutical applications. This uncertainty underscores the complexities of interpreting Theophylline’s origin in metabolic studies and highlights the need for further research to distinguish between these potential sources. VMA is closely related to neurotransmitter metabolism, particularly in oxidative stress defense (Lee et al., 2017). Oxidative stress has been confirmed to play a crucial role in AD pathology (Bai et al., 2022), and VMA may influence neuronal health by regulating oxidative stress balance. Previous studies have found that VMA levels are significantly elevated in the CSF of AD patients (Kaddurah-Daouk et al., 2013). Adenosine, a neuromodulator, regulates neurotransmitter release and inflammatory responses through the activation of its coupled receptors (Borea et al., 2018). Dysregulation of the Adenosine system in AD patients may accelerate neurotransmitter imbalances and inflammatory responses (Chang et al., 2021; Trinh et al., 2022). The dynamic differences in these metabolites not only provide earlier warning signals for CN individuals but also offer critical clues for identifying metabolic differences in the early progression of AD, which is of significant clinical importance.

Furthermore, at the key transition point of AD progression, 1,7-Dimethyluric Acid, Cystathionine, and Indole demonstrated strong predictive potential. First, 1,7-Dimethyluric Acid was significantly expressed in the early stages of AD progression, possibly reflecting its protective role in counteracting neurodegenerative differences . However, as AD progresses, 1,7-Dimethyluric Acid levels significantly decrease in the later stages of the disease, suggesting that its protective effects may diminish or disappear. 1,7-Dimethyluric Acid is one of the metabolites of caffeine, and studies have shown that caffeine and its metabolites can protect against AD through antioxidant, anti-inflammatory, and Adenosine receptor-mediated mechanisms (Fu et al., 2023; Iranpour et al., 2020; Takeshige-Amano et al., 2020). Secondly, Cystathionine was significantly expressed in the early stages of AD, possibly reflecting enhanced metabolic defense mechanisms. However, its rapid decline as the disease progresses may indicate a weakening of the antioxidant defense system, exacerbating AD progression. Cystathionine is closely associated with oxidative stress and Glutathione metabolism, processes that play important roles in AD pathology (Korczowska-Ła̧cka et al., 2023; Xi et al., 2023). Previous studies have found significant differential expression of Cystathionine in both AD and Parkinson’s disease patients (Kalecký et al., 2022), and its decline may reflect increased oxidative damage (Haddad et al., 2021; Korczowska-Ła̧cka et al., 2023). Finally, Indole was found to be metabolically suppressed in the early stages of AD but significantly increased in the later stages, possibly related to the activation of late-stage inflammatory or immune responses. Indole is an important metabolite of Tryptophan metabolism, involved in regulating the immune system, neuroprotection, and the interaction between the gut microbiota and the nervous system (Zhou et al., 2023). Previous research has identified Indole as a potential target for AD treatment (Azmy et al., 2023; Zhou et al., 2023), and this study further reveals the dynamic differences of Indole during the transition to AD.

Monitoring the dynamic differences of metabolites during AD progression is crucial for early prediction and intervention. However, this study has certain limitations. The relatively small sample size may not adequately represent the broader population, potentially leading to variations that could impact the generalizability of our findings. Additionally, the lack of controlled conditions for urine collection, such as specific collection times and dietary restrictions prior to sampling, might introduce variability that could affect the observed metabolic profiles. Moreover, this study did not account for the potential effects of medications, particularly cholinesterase inhibitors, which are commonly prescribed to manage symptoms of AD. The absence of controls for medication use represents a significant limitation, as such treatments could influence the metabolic profiles observed. Future research would benefit from addressing these factors, potentially including comprehensive data on medication usage to better understand its impact on metabolic changes associated with AD progression. Finally, although the AD progression prediction model demonstrated strong classification abilities, it requires further validation with larger clinical datasets to confirm its efficacy and robustness.

In conclusion, this study deepens the understanding of dynamic metabolic differences during AD progression, providing a foundation for future research and clinical applications. Further studies should validate these findings in larger cohorts and explore the specific roles of these metabolites in the pathogenesis of AD.

## Conclusion

This study conducted a comprehensive analysis of the metabolic differences in key metabolites and pathways at various stages of AD progression, highlighting their potential as biomarkers for early detection and intervention. The development of an AD progression prediction model has further validated the effectiveness of these biomarkers. By establishing these metabolites and pathways as reliable indicators, this research provides critical insights into the early stages of Alzheimer’s disease and lays the foundation for the development of more targeted and effective intervention strategies.

## Data Availability

Publicly available datasets were analyzed in this study. This data can be found at: https://www.genome.jp/kegg/; https://hmdb.ca/.
