# Supplementary figures and images for: Untargeted urine metabolomics reveals dynamic metabolic differences and key biomarkers across different stages of Alzheimer’s disease
Source: Front Aging Neurosci. 2025 Jan 27;17:1530046. doi: 10.3389/fnagi.2025.1530046 (PMC11807997; doi:10.3389/fnagi.2025.1530046)

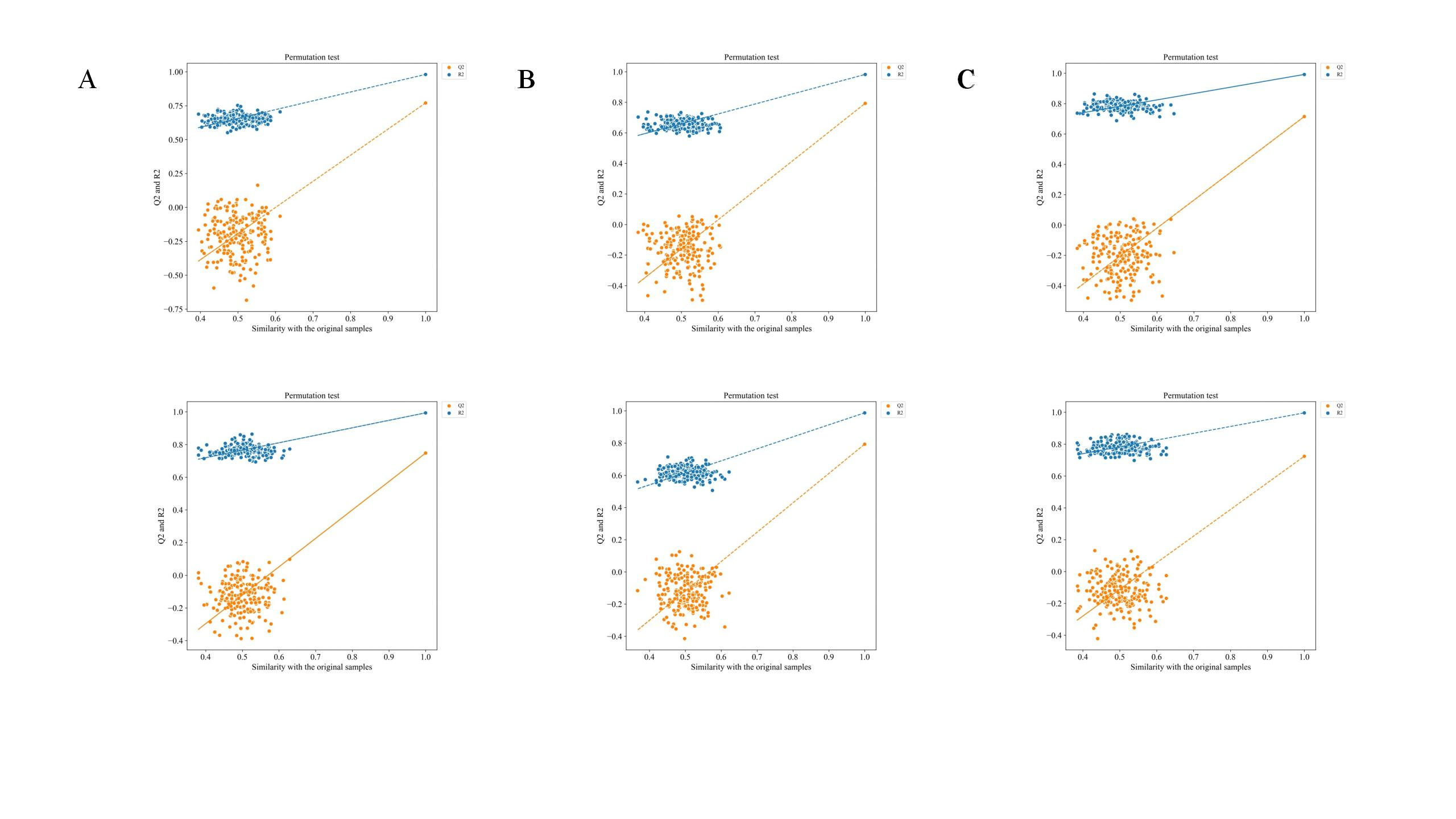

Supplement: Supplementary file 1 [file Image_1.tif]

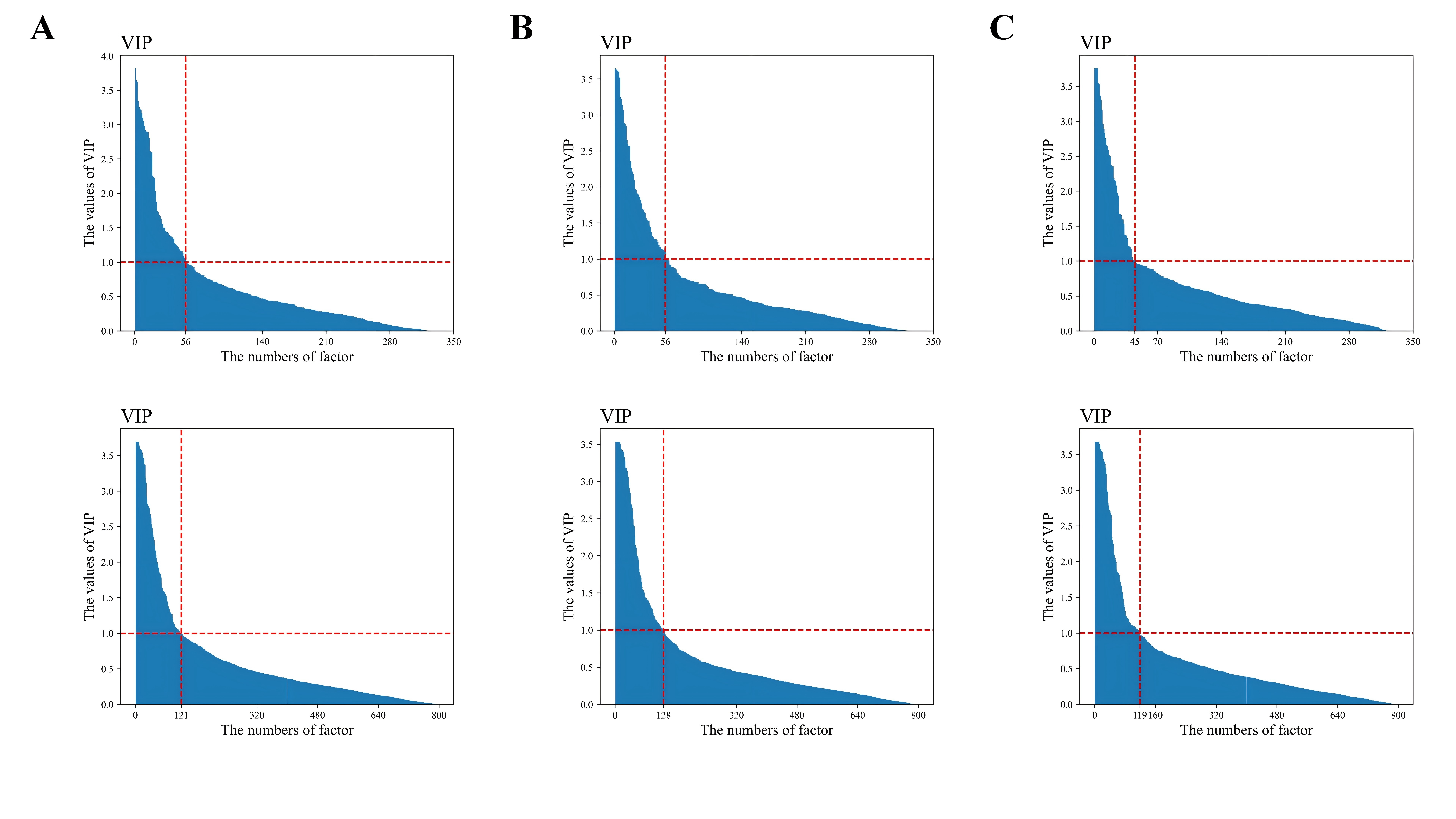

Supplement: Supplementary file 2 [file Image_2.tif]

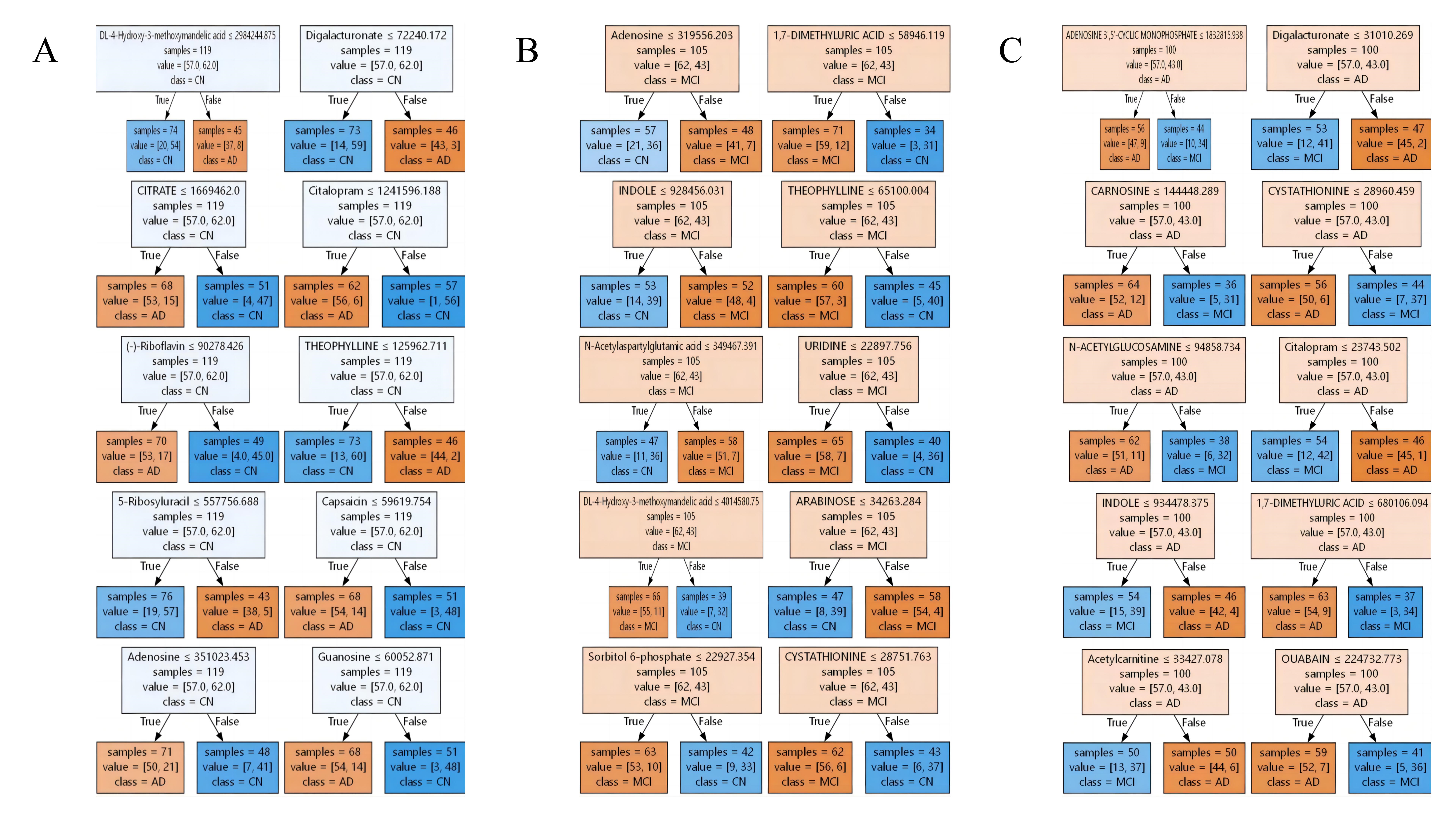

Supplement: Supplementary file 3 [file Image_3.tif]

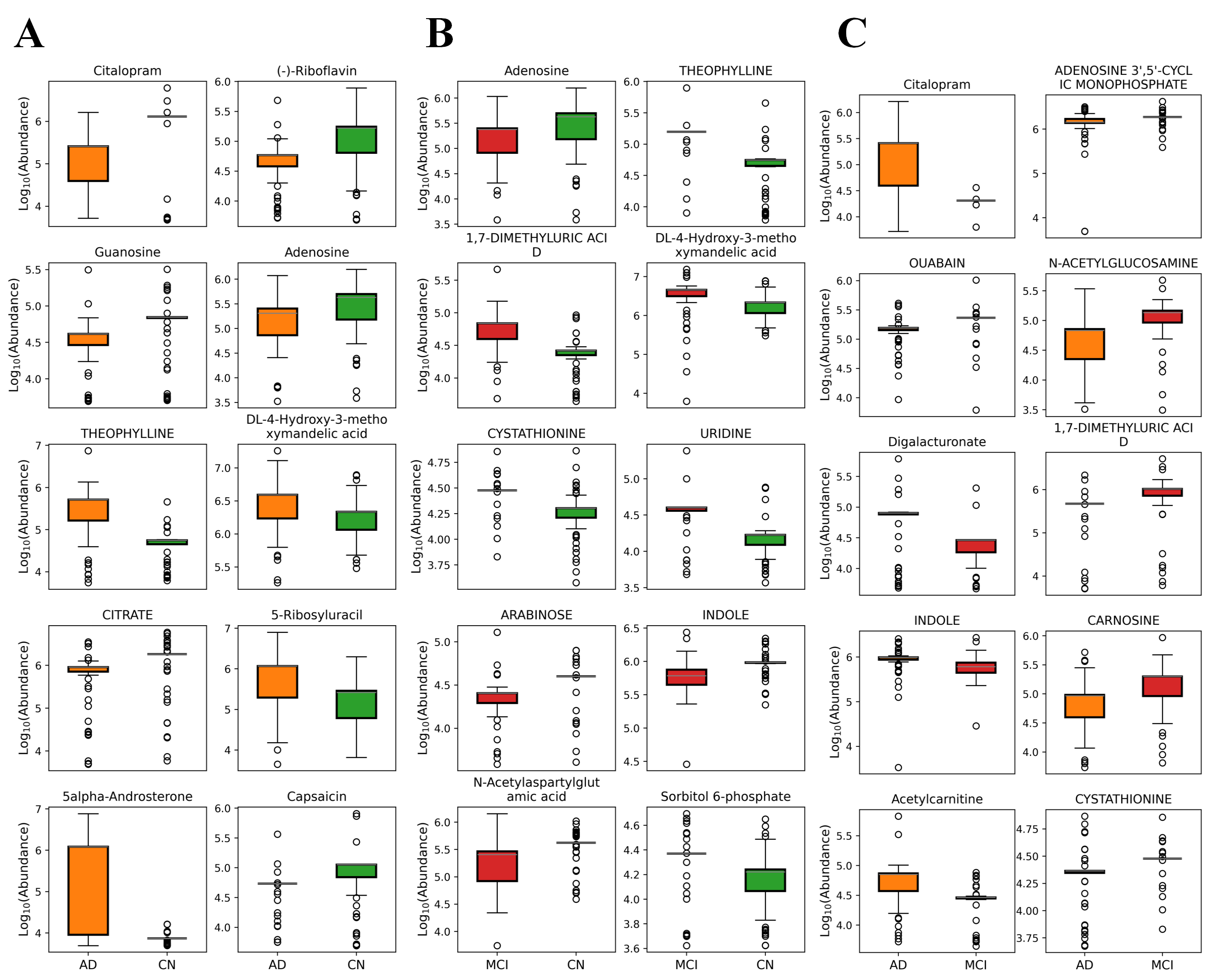

Supplement: Supplementary file 4 [file Image_4.tif]
